# Ethanol Extract of *Liriope platyphylla* Root Attenuates Non-Alcoholic Fatty Liver Disease in High-Fat Diet-Induced Obese Mice via Regulation of Lipogenesis and Lipid Uptake

**DOI:** 10.3390/nu13103338

**Published:** 2021-09-24

**Authors:** Trang Nu Huyen Le, Ho-Jung Choi, Hee-Sook Jun

**Affiliations:** 1 Gachon Institute of Pharmaceutical Science, College of Pharmacy, Gachon University, 191 Hambakmoe-ro, Yeonsu-gu, Incheon 21936, Korea; lenuhuyentrang@gmail.com (T.N.H.L.); hchoi@gachon.ac.kr (H.-J.C.); 2 Lee Gil Ya Cancer and Diabetes Institute, Gachon University, 155 Gaetbeol-ro, Yeonsu-gu, Incheon 21999, Korea; 3 Gachon Medical Research Institute, Gil Hospital, 21 Namdong-daero 774beon-gil, Namdong-gu, Incheon 21565, Korea

**Keywords:** *Liriope platyphylla*, NAFLD, lipogenesis, lipid uptake

## Abstract

Non-alcoholic fatty liver disease (NAFLD) is a common metabolic disorder that causes excess lipid accumulation in the liver and is the leading cause of end-stage liver disease. *Liriope platyphylla* is a medicinal herb that has long been used to treat cough, obesity, and diabetes. However, the effect of *Liriope platyphylla* on NAFLD has not been studied. The aim of this study was to investigate the effect of *Liriope platyphylla* root ethanolic extract (LPE) on hepatic lipid accumulation in high-fat diet (HFD)-induced obese mice. Six-week-old C57BL/6 male mice were fed a HFD for 8 weeks and then treated with LPE (100 or 250 mg/kg/day) by oral gavage for another 8 weeks. Body weight gain and liver weight were significantly lower in the 250 mg/kg LPE-treated HFD group than in the vehicle-treated HFD group. Histological analysis of liver sections demonstrated that LPE treatment reduced lipid accumulation compared to the vehicle treatment. The serum total cholesterol, AST, and ALT levels significantly decreased in the LPE-treated HFD group compared to those in the vehicle-treated HFD group. The LPE significantly decreases the protein expression levels of SREBP1, ACC, p-ACC, FAS, and SCD1, which are involved in lipogenesis, and PPARγ, CD36/FAT, and FATP5, which are involved in fatty acid uptake, both in vivo and in vitro. Thus, LPE may attenuate HFD-induced NAFLD by decreasing lipid accumulation by inhibiting lipogenesis and fatty acid uptake.

## 1. Introduction

The increase in obesity and type 2 diabetes worldwide has led to a corresponding increase in the incidence of non-alcoholic fatty liver disease (NAFLD) [1]. NAFLD is characterized by excessive lipid accumulation in the liver, which can easily progress from simple steatosis to non-alcoholic steatohepatitis (NASH) and hepatocellular carcinoma [2]. Several studies have attempted to develop novel therapies for NAFLD based on diverse molecular mechanisms, such as the farnesoid X receptor agonist, the peroxisome proliferator-activated receptor (PPAR) agonist, and the caspase inhibitor [3]. However, no medication has been approved by the U.S. Food and Drug Administration for the treatment of NAFLD [4]. Many studies have explored novel medicines for the treatment of NAFLD using herbal plants because they have fewer side effects [3,5,6]. 

It is known that hepatic dyslipidemia is a key contributor to the development of NAFLD. It is induced by obesity, type 2 diabetes, insulin resistance, inflammation, and oxidative stress [7,8]. Hepatic lipid accumulation is affected by de novo lipogenesis (DNL), uptake of circulating lipids, and fatty acid oxidation [9,10]. The transcriptional regulation of DNL is regulated by sterol regulatory element-binding protein 1c (SREBP1c). Increased expression of SREBP1c protein stimulates the expression of acetyl-CoA carboxylase (ACC), fatty acid synthase (FAS), and stearoyl-CoA desaturase-1 (SCD1), which are downstream target genes [11,12]. DNL contributes to hepatic steatosis and steatohepatitis by synthesizing saturated fatty acids, such as palmitate, which can induce inflammation and apoptosis [13]. The excessive influx of fatty acids to the liver is one of the causes of NAFLD development, and this influx is mediated by fatty acid transport proteins (FATP) and cluster of differentiation 36/fatty acid translocase (CD36/FAT) on the hepatocyte plasma membrane [14]. FATP and CD36 are known to be regulated by PPARγ because the PPAR response element is present in their promoters [15,16]. A decrease in hepatic β-oxidation is also a contributing factor to hepatic lipid accumulation [17,18]. Fatty acid oxidation occurs mainly in the mitochondria, peroxisomes, and cytochromes, and is regulated by several genes, such as carnitine palmitoyltransferase 1 (CPT1) and peroxisomal acyl-coenzyme A oxidase 1 (ACOX1) [11]. 

*Liriope platyphylla* is an herbal drug with various pharmacological and biological properties, such as anti-obesity, anti-diabetes, and anti-inflammatory effects [19,20,21,22]. It has also been reported to inhibit abdominal fat accumulation and improve glucose regulation in Otsuka Long-Evans Tokushima Fatty type II diabetes rats [23]. In 3T3-L1 adipocytes, insulin-stimulated glucose uptake was increased by the homoisoflavone-enriched fraction of *L. platyphylla* [21]. In this study, we focused on the effect of *L. platyphylla* root ethanol extract (LPE) on hepatic lipid accumulation in high-fat diet (HFD)-induced obese mice to determine whether LPE could potentially be used to therapeutically treat NAFLD.

## 2. Materials and Methods

### 2.1. LPE Preparation

In this study, we used a 70% (*v*/*v*) ethanolic extract of dried *L. platyphylla* root. The freeze-dried LPE powder (KOC-70E-287) was obtained from KOC Biotech (Yuseong-gu, Daejeon, South Korea). The dried ethanol extract was suspended in phosphate-buffered saline (PBS) for the in vivo experiments. For the in vitro experiment, the dried ethanol extract was suspended in DMSO to a concentration of 100 mg/mL and stored at −20 °C.

### 2.2. Animals and Experimental Design

Six-week-old C57BL/6 male mice were obtained from OrientBio (Seongnam-si, Kyunggido, South Korea). After 1 week of acclimation, the mice were fed either a HFD (60% fat primarily from lard, Research Diets, Inc., New Brunswick, NJ, USA, #D12492) or a normal chow diet (5.4% fat) for 8 weeks to establish the HFD-induced obese model. The mice were randomly divided into four groups: the normal chow group (control; *n* = 7), a PBS-treated HFD group (HFD; *n* = 11), a 100 mg/kg LPE-treated HFD group (HFD_LPE_100), and a 250 mg/kg LPE-treated group (HFD_LPE_250). The LPE was dissolved in PBS and the mice received either PBS (control and HFD) or 100 or 250 mg/kg LPE (HFD_LPE_100 or HFD_LPE_250 in PBS) by oral gavage for another 8 weeks. Body weights were measured daily. Subsequently, the mice were sacrificed, and blood and liver tissues were collected, immediately weighed, and stored at −80 °C. All animal experiments were performed in compliance with the ethical requirements of the Laboratory Animal Research Center, College of Pharmacy, Gachon University, Korea. The experimental protocol was approved by the Gachon University Institutional Animal Care and Use Committee (GIACUC-R2018012). 

### 2.3. Serum Biochemical Analyses 

After 8 weeks of LPE treatment, blood samples were taken from the tail veins of the mice and centrifuged at 12,000 rpm for 30 min at 4 °C to obtain serum samples. The serum levels for total cholesterol (TC), triglyceride (TG), low-density lipoprotein (LDL) cholesterol, aspartate aminotransferase (AST), and alanine aminotransferase (ALT) were analyzed by the Dongnam Medical Research Institute (Suyeong-gu, Busan, South Korea). 

### 2.4. Histological Staining and NAFLD Activity Score (NAS)

For hematoxylin and eosin (H&E) staining, the tissues were first fixed in a 10% neutral buffered formalin solution. Then, they were embedded in paraffin, cut into 5 μm-thick sections, and stained with hematoxylin and eosin. For Oil Red O staining, the liver tissue was fixed in an optimal cutting temperature compound (Sakura, Torrance, CA, USA), cut into 10 μm-thick sections, and then stained with Oil Red O and Mayer’s hematoxylin solution for microscopy [24]. The stained sections were observed and photographed using a confocal microscope (Nikon Intensilight C-HGFI, Tokyo, Japan).

Steatosis, inflammation, and ballooning were graded according to the NAS criteria [25]. NAS includes the punctuation of steatosis, inflammation, ballooning, and fibrosis in routinely stained liver sections. All images were analyzed with ImageJ software (NIH, Bethesda, MD, USA). 

### 2.5. Cell Culture and Cell Viability Assay

Human hepatocellular carcinoma HepG2 cells were maintained in DMEM (Welgene, Gyeongsangbuk-do, Korea) supplemented with 10% fetal bovine serum (Welgene, Gyeongsangbuk-do, Korea) and 1% antibiotics (100 U/mL penicillin and 100 μg/mL streptomycin) (Welgene, Gyeongsangbuk-do, Korea) at 37 °C in a humidified 5% CO_2_ atmosphere. HepG2 cells were seeded at 1 × 10^4^ cells/well in 96-well plates and treated with various concentrations of LPE or free fatty acid (FFA) in a growth medium for 24 h. The FFA was mixed with two volumes of oleic acid and one volume of palmitic acid and then conjugated with 4% bovine serum albumin (BSA) to a final concentration of 15 mM FFA mixture containing 1% BSA. The BSA-conjugated FFA mixture solution was diluted in the growth medium and used for the experiment. After 1 day of treatment, Cell Counting Kit-8 (CCK-8) solution was added, the mix was incubated at 37 °C for 2 h, and the absorbance was recorded at 450 nm using a microplate reader (VersaMax, Molecular Devices).

### 2.6. Western Blot Analysis

HepG2 cells were seeded at 2.5 × 10^5^ cells/well in 6-well plates and treated with 50 or 100 μg/mL of LPE or 0.25 mM of FFA in growth medium for 12 h. Total protein from the liver tissues or HepG2 cells was extracted in a mixture of mammalian protein extract buffer (GE Healthcare Life Sciences, Marlborough, MA, USA), protease inhibitor, and phosphatase inhibitor (Sigma-Aldrich, St Louis, MO, USA). Twenty micrograms of the lysates were separated using sodium dodecyl sulfate-polyacrylamide gel electrophoresis. Then, nitrocellulose membranes (GE Healthcare Life Sciences, Marlborough, MA, USA) were used for the transfer and 5% BSA, and Fraction V (MP Biomedicals, Irvine, CA, USA) was used for 1 h to block the transferred membrane, which was then incubated with primary antibodies: FAS and SCD1 (Santa Cruz Biotechnology, Paso Robles, CA, USA), SREBP1, FATP5, and CD36 (Abcam, MA, USA), and ACC, p-ACC, and β-actin (Cell Signaling, Danvers, MA, USA). The membrane was then incubated with secondary antibodies for 1 h at room temperature. The protein bands were visualized using enhanced chemiluminescence (ECL) detection kits and detected using the Chemidoc^TM^ XRS^+^ system. ImageJ software was used to quantify the optical density of the protein bands.

### 2.7. Measurement of Hepatic Triglyceride from Liver Tissue and HepG2 Cells 

Five mg of isolated liver tissue was used to isolate the TG. HepG2 cells were seeded at 2.5 × 10^5^ cells/well in 6-well plates and treated with 50 or 100 μg/mL of LPE or 0.25 mM of FFA in growth medium for 24 h. The cells were harvested and then counted for normalization. The TG was isolated from the liver tissue and HepG2 cells using the Folch method [26]. Briefly, 20 times more chloroform/methanol (2:1 *v*/*v*) was added compared to the amount of liver tissue. The tissues were ground and shaken for 20 min at room temperature and then the mixture was centrifuged at 12,000 rpm for 20 min. After removing the liquid phase, 0.2% NaCl (0.9%) was added. Then, the mixture was vortexed, centrifuged at 2000 rpm for 5 min, and the lower layer was removed and placed in a new tube. The lower layer solution was dried with a chemical hood. After drying, 100% isopropanol was added to the mixture. The TG concentration was measured using a TG reagent kit (Asan Pharmaceutical, Seoul, South Korea) according to the manufacturer’s instructions. Cell numbers and tissue weights were used for normalization after the TG from HepG2 cells and the liver tissue had been measured.

### 2.8. Statistical Analysis

Data are presented as the mean ± SEM. The statistical analysis was performed using an unpaired parametric ANOVA followed by Fisher’s protected least significant difference test for multiple groups. * *p* < 0.05 was considered significant.

## 3. Results

### 3.1. LPE Treatment Decreased Body Weight Gain and Hepatic Lipid Accumulation

**Figure 1:**
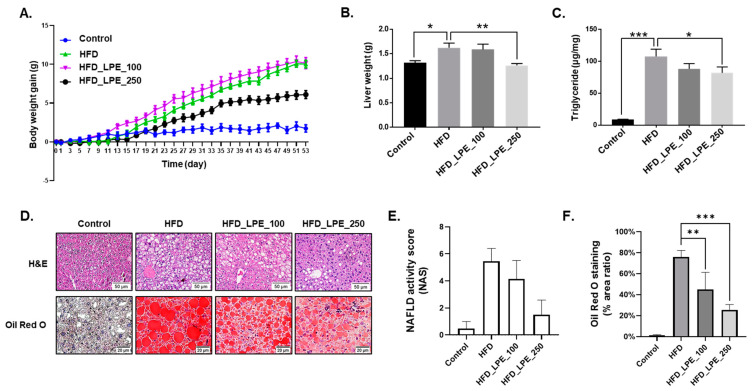
*Liriope platyphylla* root ethanolic extract (LPE) (250 mg/kg BW) treatment decreased body weight gain and hepatic lipid accumulation. After 8 weeks of high fat diet (HFD) feeding, C57BL/6 male mice were administered LPE (100 or 250 mg/kg/day) by oral gavage for 8 weeks. The HFD-fed mice were continuously fed 60% HFD during the experimental period. (**A**) Changes in body weight were monitored daily. (**B**) The liver was collected and weighed after 8 weeks of LPE treatment. (**C**) The liver triglyceride (TG) levels were measured. Data are shown as mean ± standard error of the mean (SEM), *n* = 6–11/group. (**D**) Hematoxylin and eosin (H&E) and Oil Red O staining of liver sections (magnification, ×200). (**E**) Non-alcoholic fatty liver disease (NAFLD) activity score (NAS). (**F**) The area ratio (%) of Oil Red O staining was measured with ImageJ software. Data are shown as mean ± standard deviation (SD), *n* = 5/group. * *p* < 0.05, ** *p* < 0.01, or *** *p* < 0.001. One-way ANOVA was performed along with Fisher’s protected least significant difference test.

To investigate whether LPE has an effect on weight loss in HFD-induced obese mice, the mice were orally administered 100 or 250 mg/kg of LPE daily for 8 weeks. As shown in Figure 1A, the HFD-fed mice (HFD group) showed significantly increased body weight gain compared to mice fed a normal diet (control group). The body weight gain of the 100 mg/kg LPE-administered mice (HFD_LPE_100 group) was not significantly different from that of the HFD group. However, the body weight gain of the mice treated with 250 mg/kg of LPE (HFD_LPE_250 group) was significantly reduced compared to the HFD group. We checked the weight of the liver after 8 weeks of LPE administration. The liver weight was higher in the HFD group than in the control group, but there were no differences in liver weight between the HFD group and the HFD_LPE_100 group. The liver weight was remarkably decreased in the HFD_LPE_250 group compared to that in the HFD group (Figure 1B). 

In numerous studies, HFD-induced obese mice showed remarkably increased hepatic TG levels [27,28]. We measured TG levels in liver tissues to examine whether treatment with LPE affects hepatic lipid content in HFD-induced obese mice. Hepatic TG levels in the HFD group were significantly higher than those in normal mice. Treatment with 250 mg/kg of LPE significantly suppressed the HFD-induced increase in hepatic TG levels (Figure 1C). Hematoxylin and eosin staining of paraffin sections was performed to check lipid accumulation in the tissue samples. The HFD group showed cytoplasmic vacuolization, which suggests the presence of lipids in the liver. The cytoplasmic vacuolization in the HFD_LPE_100 group was not significantly different to that in the HFD group. However, cytoplasmic vacuolization in the HFD_LPE_250 group had obviously decreased compared to that in the HFD group (Figure 1D). We evaluated the NAS using H&E-stained slides. The HFD_LPE_250 group showed a lower NAS score than the HFD group (Figure 1E). We performed Oil Red O staining using frozen sections to confirm the lipid content in the liver. Lipid droplets stained red by Oil Red O obviously increased in the HFD group compared to the control group. Lipid droplet accumulation was reduced in the HFD_LPE_250 group compared to that in the HFD group (Figure 1D,F). 

### 3.2. LPE Treatment Improved Liver Injury and Serum Lipid Levels in HFD Mice

**Figure 2:**
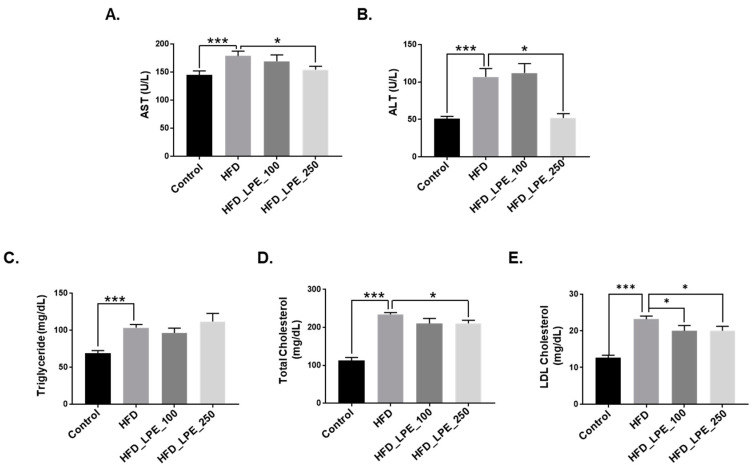
Effect of LPE on serum biochemical parameters in HFD-fed mice. Eight weeks after beginning the HFD, the C57BL/6 mice were orally administered LPE (100 or 250 mg/kg body weight) or PBS daily. Blood samples were collected after 8 weeks, and their biochemical parameters were measured. (**A**) Aspartate aminotransferase (AST), (**B**) alanine aminotransferase (ALT), (**C**) total triglyceride, (**D**) total cholesterol, and (**E**) low-density lipoprotein (LDL) cholesterol. Data are shown as mean ± SEM, *n* = 7–11/group, * *p* < 0.05, *** *p* < 0.001. One-way ANOVA was performed along with Fisher’s protected least significant difference test.

Hepatocellular injury is caused by lipid accumulation in the liver [29]. Therefore, serum ALT and AST levels were measured to evaluate whether LPE treatment improves liver injury. Serum AST and ALT levels decreased in the HFD_LPE_250 group compared to the HFD group; however, serum AST and ALT levels did not change between the HFD group and the HFD_LPE_100 group (Figure 2A,B). Next, we analyzed the serum parameters, including serum TG and cholesterol levels. Serum TG, TC, and LDL cholesterol levels were significantly higher in the HFD group than in the control group (Figure 2C–E). Serum TG levels were not changed by the LPE treatment, but serum TC levels were significantly lower in the HFD_LPE_250 group than in the HFD group (Figure 2D). Furthermore, serum LDL cholesterol levels significantly decreased in the HFD_LPE_100 and HFD_LPE_250 groups (Figure 2E). 

### 3.3. LPE Treatment Decreased the Expression of Proteins Involved in Lipogenesis in the Livers of HFD-Fed Mice

**Figure 3:**
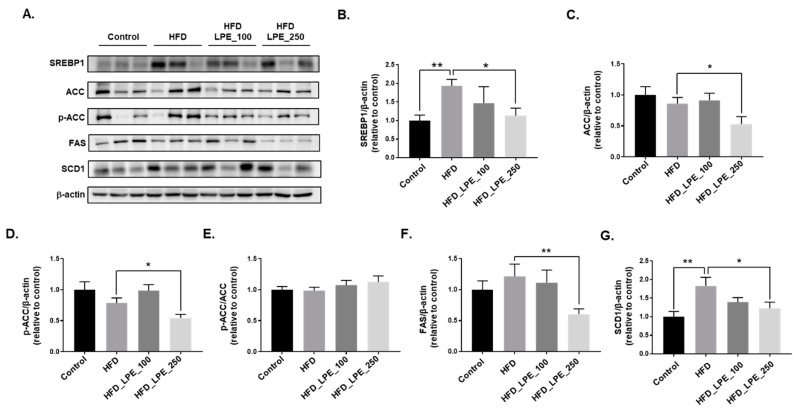
LPE treatment decreased the expression of proteins involved in lipogenesis in HFD-fed mice. SREBP1, ACC, p-ACC, FAS, and SCD1 protein expression levels were analyzed using Western blotting. Beta-actin was used as the loading control for equal amounts of protein. (**A**) Representative immunoblots of SREBP1, ACC, p-ACC, FAS, and SCD1, together with β-actin, and the relative intensity levels of (**B**) SREBP1, (**C**) ACC, (**D**) p-ACC, (**E**) p-ACC/ACC, (**F**) FAS, and (**G**) SCD1, were normalized to beta-actin. Data are shown as mean ± SEM, *n* = 6–8/group for SCD1, *n* = 6–11/group for other genes. * *p* < 0.05 or ** *p* < 0.01. One-way ANOVA was performed along with Fisher’s protected least significant difference test.

Figure 1C demonstrates that LPE attenuates hepatic lipid accumulation in the HFD_LPE_250 group compared to the HFD group. Hepatic lipid accumulation is regulated by several pathways, such as the lipogenesis, fatty acid uptake, and beta-oxidation pathways [11]. To investigate whether LPE treatment controls hepatic lipogenic gene expression, we analyzed the protein expression levels of SREBP1, SCD1, FAS, p-ACC, and ACC. SREBP1 is a major regulator of hepatic lipogenesis and transactivates lipogenic genes, such as ACC, FAS, and SCD1 [11]. The SCD1 protein level was significantly higher in the HFD group than in the control group. In contrast, the SREBP1, SCD1, FAS, p-ACC, and ACC protein levels were significantly reduced in the HFD_LPE_250 group compared to the HFD group. However, p-ACC/ACC was not changed by LPE administration (Figure 3). These data indicated that LPE decreased lipogenesis through inhibition of the SREBP1 pathway. 

### 3.4. LPE Treatment Decreased the Expression of Proteins Involved in Fatty Acid Uptake in the Livers of HFD-Fed Mice

**Figure 4:**
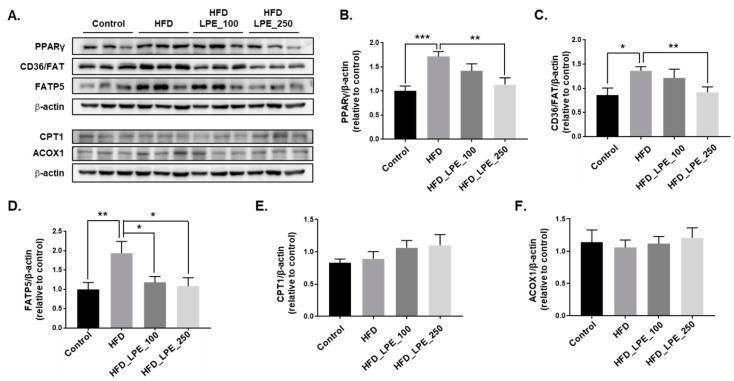
LPE treatment decreased the expression of proteins involved in fatty acid uptake in HFD-fed mice. PPARγ, CD36/FAT, FATP5, CPT1, and ACOX1 protein expressions were analyzed by Western blotting. Beta-actin was used as the loading control for equal amounts of protein. (**A**) Representative immunoblots of PPARγ, CD36/FAT, FATP5, CPT1, and ACOX1, together with β-actin, and the relative intensity levels of (**B**) PPARγ, (**C**) CD36/FAT, (**D**) FATP5, (**E**) CPT1, and (**F**) ACOX1, were normalized to beta-actin. Data are shown as mean ± SEM, *n* = 6–11/group. * *p* < 0.05, ** *p* < 0.01, or *** *p* < 0.001. One-way ANOVA was performed along with Fisher’s protected least significant difference test.

We analyzed the protein expression levels of PPARγ, CD36/FAT, and FATP5 to evaluate whether LPE treatment attenuates hepatic fatty acid uptake. CD36/FAT and FATP5 are well-known fatty acid transport proteins in the liver [11], and PPARγ is a transcription factor that induces the mRNA expression of CD36/FAT and FATPs [15,16]. PPARγ, CD36/FAT, and FATP5 protein expression levels significantly increased in the HFD group compared to the control group, whereas their levels significantly decreased in the HFD_LPE_250 group compared to the HFD group (Figure 4A–D). 

We analyzed the protein expression levels of CPT1 and ACOX1 to evaluate whether the LPE treatment increases fatty acid β-oxidation. Studies show that CPT1 and ACOX1 induce β-oxidation [30,31]. CPT1 and ACOX1 protein expression levels were not significantly changed in the LPE administration groups compared to the HFD group (Figure 4E,F).

### 3.5. LPE Treatment Decreased TG Accumulation in FFA-Treated HepG2 Cells

**Figure 5:**
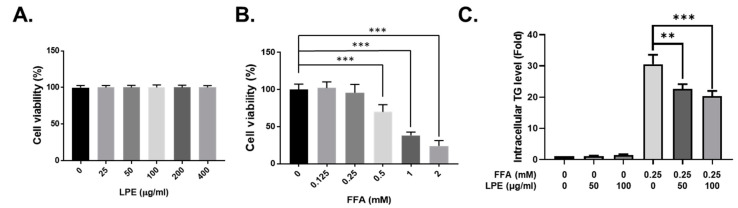
LPE treatment decreased TG accumulation in FFA-treated HepG2 cells. (**A**) Effects of LPE on cell viability in HepG2 cells. HepG2 cells were incubated with various concentrations of LPE for 24 h and the cell viability was measured using a CCK-8 assay kit. (**B**) Effects of FFA on HepG2 cell viability. HepG2 cells were incubated with various concentrations of FFA for 24 h and the cell viability was measured using a CCK-8 assay kit. (**C**) Effect of LPE on FFA-induced TG accumulation in HepG2 cells. HepG2 cells were incubated with or without 0.25 mM of FFA and 50 or 100 µg/mL of LPE for 24 h. The TG was measured using a TG assay kit. Data are shown as mean ± SEM, *n* = 3, ** *p* < 0.01 or *** *p* < 0.001. One-way ANOVA was performed along with Fisher’s protected least significant difference test.

We used the HepG2 cell line to investigate the direct effect of LPE on hepatic lipid accumulation. We first checked whether LPE had cytotoxic effects on HepG2 cells. The LPE treatments were 0, 25, 50, 100, 200, or 400 µg/mL for 24 h. No cytotoxicity was observed after LPE treatment (Figure 5A). To investigate hepatic steatosis in vitro, a cellular model was established in HepG2 cells with FFA solution, which contained palmitate and oleate [32]. The HepG2 cells were incubated with FFA mixtures (1 volume of palmitate and 2 volumes of oleate) up to 2 mM for 24 h. The 0.125 or 0.25 mM FFA concentrations did not affect cell viability (Figure 5B). Therefore, we treated the HepG2 cells with 0.25 mM of FFA and 50 or 100 µg/mL of LPE for 24 h to induce hepatic steatosis. The FFA-induced HepG2 cells showed significant increases in lipid accumulation, but LPE at a dose of 50 or 100 µg/mL significantly inhibited this increase (Figure 5C).

### 3.6. LPE Treatment Decreased the Expression of Proteins Involved in Lipogenesis in FFA-Treated HepG2 Cells

**Figure 6:**
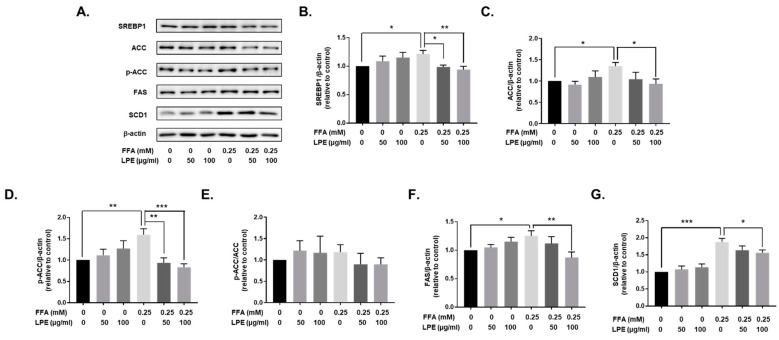
LPE treatment decreased the expression of proteins involved in lipogenesis in FFA-treated HepG2 cells. SREBP1, ACC, p-ACC, FAS, and SCD1 protein expressions were analyzed by Western blotting. Beta-actin was used as the loading control for equal amounts of protein. (**A**) Representative immunoblots of SREBP1, ACC, p-ACC, FAS, and SCD1, together with the relative intensity levels of (**B**) SREBP1, (**C**) ACC, (**D**) p-ACC, (**E**) p-ACC/ACC, (**F**) FAS, and (**G**) SCD1, were normalized to β-actin. Data are shown as mean ± SEM, *n* = 5/group, * *p* < 0.05, ** *p* < 0.01, *** *p* < 0.001. One-way ANOVA was performed along with Fisher’s protected least significant difference test.

We treated HepG2 cell line cells with 0.25 mM of FFA and 50 or 100 µg/mL of LPE for 12 h to confirm whether LPE treatment changes the expression of molecules related to lipogenesis and fatty acid uptake, as observed in ex vivo liver tissues from LPE-treated HFD-fed mice. Treatment with 0.25 mM of FFA for 12 h significantly enhanced SREBP1, ACC, p-ACC, FAS, and SCD1 expression in HepG2 cells. Treatment with 100 µg/mL of LPE significantly reduced SREBP1, ACC, p-ACC, FAS, and SCD1 expression compared to FFA-treated HepG2 cells. p-ACC/ACC was not changed by LPE treatment (Figure 6).

### 3.7. LPE Treatment Decreased the Expression of Proteins Involved in Fatty Acid Uptake in FFA-Treated HepG2 Cells

**Figure 7:**
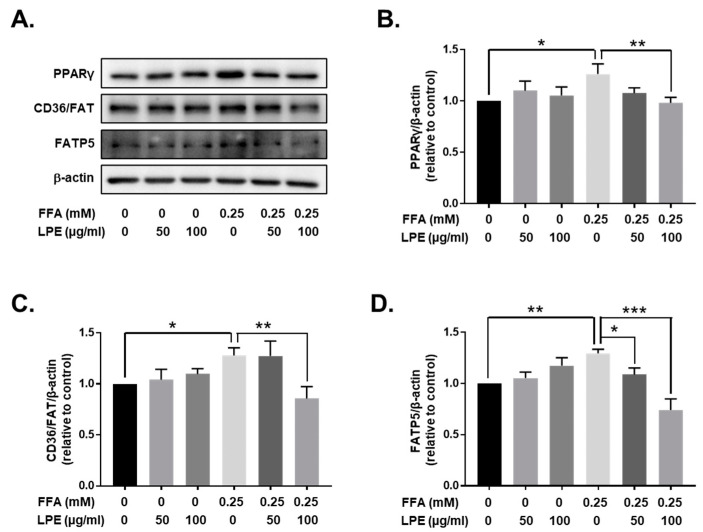
LPE treatment decreased the expression of proteins involved in fatty acid uptake in FFA-treated HepG2 cells. PPARγ, CD36/FAT, and FATP5 protein expressions were analyzed by Western blotting. Beta-actin was used as the loading control for equal amounts of protein. (**A**) Representative immunoblots of PPARγ, CD36/FAT, and FATP5, together with the relative intensity levels of (**B**) PPARγ, (**C**) CD36/FAT, and (**D**) FATP5, were normalized to beta-actin. Data are shown as mean ± SEM, *n* = 5/group, * *p* < 0.05, ** *p* < 0.01, *** *p* < 0.001. One-way ANOVA was performed along with Fisher’s protected least significant difference test.

In addition, we investigated the expression of proteins involved in fatty acid uptake, such as CD36/FAT and FATP5, in HepG2 cells. The FFA-treated HepG2 cells exhibited higher CD36/FAT and FATP5 protein expressions. Treatment with LPE (100 µg/mL) significantly attenuated the expression of these proteins compared to those in FFA-treated HepG2 cells (Figure 7). In addition, PPARγ expression, the transcription factor for CD36/FAT and FATP5, was also inhibited in HepG2 cells treated with LPE. These results confirmed that LPE treatment also reduced the expression of proteins involved in lipogenesis and fatty acid uptake in vitro. 

## 4. Discussion

NAFLD is one of the most common causes of chronic liver diseases [33]. Metabolic syndromes, such as obesity, type 2 diabetes mellitus, and insulin resistance, are strong risk factors for the development of NAFLD [34]. Currently, the most effective management of NAFLD is weight loss through diet and physical exercise. Currently, there is no FDA-approved medicine for NAFLD, which means that there is an urgent need to develop therapies for this disease. Several natural products have been considered as alternative treatments to prevent NAFLD or to stop its progression [35,36,37]. Several studies have demonstrated the possibility of ameliorating inflammation and obesity by the administration of LPE or LPE-containing traditional medicine [20,21,22]. However, there is currently no evidence that LPE has any effect on hepatic lipid accumulation in the HFD-induced NAFLD model. In this study, we demonstrated that LPE decreased hepatic lipid accumulation and protected against liver injury in a HFD-induced NAFLD mouse model and studied the mechanisms involved.

LPE administration at 250 mg/kg, but not 100 mg/kg, prevented body weight gain and liver weight in HFD-induced obese mice (Figure 1A,B), suggesting that a higher dose (250 mg/kg) had therapeutic effects. In addition, LPE administration decreased liver TG content and Oil Red O-stained lipid droplets (Figure 1C,D). Liver injury can be examined by investigating AST and ALT levels. In particular, when liver injury and hepatotoxicity occur, the serum AST and ALT levels increase, which is associated with hepatic steatosis [38]. Serum AST and ALT levels significantly increased in HFD-fed mice, but these levels were decreased by LPE treatment, demonstrating that LPE can protect mice against HFD-induced liver damage (Figure 2A,B). Dyslipidemia, which is associated with NAFLD, is defined by an abnormal quantity of lipids, such as TC, TG, and low-density lipoprotein (LDL) cholesterol in the blood [39]. Accumulating evidence suggests that lipid metabolism abnormalities, such as increased serum TG, TC, and LDL cholesterol levels, may contribute to NASH development [40]. Serum TC, TG, and LDL cholesterol levels significantly increased in the HFD group compared to those in the control group, whereas LPE administration lowered serum TC levels and LDL cholesterol (Figure 2D,E). However, serum TG levels were not affected by LPE administration (Figure 2C). Several studies have shown that hepatic and serum TG levels are not regulated in the same direction [41,42,43]. For example, Ding et al. showed that Pomelo peel extracts have a beneficial effect on HFD-induced metabolic disorders by lowering liver TG levels without decreasing serum TG levels [41]. We speculated that serum TG levels remained unchanged, owing to the decrease in fatty acid uptake and no improvement of beta-oxidation in the liver with the administration of LPE (Figure 4E,F).

The liver plays an important role in lipid metabolism processes, such as lipogenesis, fatty acid uptake, and fatty acid oxidation [11]. To understand how LPE treatment ameliorates lipid accumulation in the liver, we examined the expression of proteins involved in lipogenesis, fatty acid uptake, and fatty acid oxidation in liver tissue and FFA-treated HepG2 cells. SCD1, FAS, p-ACC, and ACC play key roles in the lipogenesis process. In lipogenesis, ACC carboxylates acetyl-CoA to malonyl-CoA, the substrate for fatty acid synthesis, and SCD1 is an important enzyme that catalyzes monounsaturated fatty acid synthesis [44]. The LPE treatment significantly decreased the expression of these proteins. In addition, the LPE treatment significantly decreased FFA-induced intracellular TG and the expression of SCD1, FAS, p-ACC, and ACC in HepG2 cells. p-ACC/ACC was not changed by the administration of LPE, suggesting that the decrease of ACC expression contributes to the decrease of phosphorylated ACC. The expression of ACC, FAS, and SCD1 is regulated by SREBP1c, which is a major regulator of lipogenesis [11]. We examined SREBP1 expression in liver tissue and HepG2 cells. The SREBP1 expression levels in HepG2 cells increased in the HFD group and after treatment with 0.25 mM of FFA. However, LPE treatment decreased SREBP1 expression. These data suggest that LPE significantly inhibited the expression of SREBP1, SCD1, FAS, p-ACC, and ACC, thereby contributing to the inhibition of TG synthesis. 

According to several studies, increased FATPs and CD36/FAT expression are linked to increased fatty acid uptake and lipid buildup in the liver, which is linked to NAFLD. As a result, fatty acid absorption factors might be potential targets for the buildup of hepatic lipids [45,46,47]. Of the six mammalian FATP isoforms, FATP2 and FATP5 are found in the liver. FATP2 is expressed in the kidney and liver, but FATP5 is only expressed in the liver [48]. Therefore, we examined the expression of CD36/FAT and FATP5. The expression of these proteins significantly increased in the HFD group compared to the control group, and LPE treatment inhibited this increase. Similar results were obtained using an in vitro model of HepG2 cells. These results suggest that LPE treatment decreased the expression of CD36 and FATP5, contributing to reduced fatty acid uptake. Accumulating evidence indicates that the overexpression of PPARγ contributes to increased hepatic lipid accumulation [49,50]. CD36 and FATP5 are expressed and regulated by PPARγ [11,50]. A HFD can induce PPARγ expression, which accelerates lipid accumulation and leads to hepatic steatosis in mouse liver cells [51]. Treatment with LPE reduced the protein expression of PPARγ both in vivo and in vitro. These data indicate that LPE inhibits fatty acid uptake by regulating PPARγ gene expression. 

An increase in fatty acid β-oxidation may be a strategy that could be used to decrease hepatic lipid accumulation [17]. In the liver, CPT1 and ACOX1 are involved in β-oxidation [52]. CPT1 is an enzyme that transports FFA into mitochondria for oxidation to produce acetyl-CoA, which is subsequently used to generate ATP via the tricarboxylic acid cycle and electron transport chain [30]. ACOX1, along with peroxidase, is an initiating enzyme in the β-oxidation system [31]. When we investigated the expressions of ACOX1 and CPT1 in liver tissue, we found that LPE treatment did not affect the expression of these proteins (Figure 4E,F). 

## 5. Conclusions

**Figure 8:**
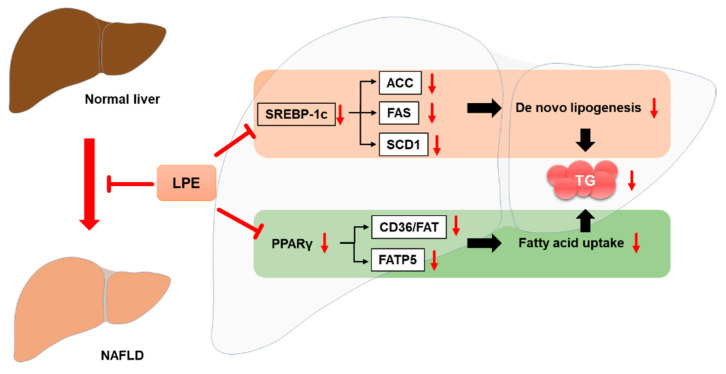
Schematic diagram of potential improvement mechanisms of HFD-induced NAFLD by LPE.

Our data suggest that LPE may attenuate HFD-induced NAFLD by decreasing lipid accumulation through inhibition of lipogenesis and fatty acid uptake, but not by an increase in fatty acid oxidation. The LPE treatment decreased the expressions of proteins involved in lipogenesis and fatty acid uptake via downregulation of SREBP1c and PPARγ, respectively (Figure 8).

## Data Availability

The data used to support the findings of this study are available from the corresponding author upon reasonable request.
